# Supplementary material for: Heparin-based hydrogel scaffolding alters the transcriptomic profile and increases the chemoresistance of MDA-MB-231 triple-negative breast cancer cells
Source: Biomater Sci. 2020 Feb 13;8(10):2786–96. doi: 10.1039/c9bm01481k (PMC7497406; doi:10.1039/c9bm01481k)
Supplement: Supplementary file 2 [file BM-008-C9BM01481K-s002.zip › Supplementary File 4/HP-B hydrogel + EGF (s)vControl/Pathways/my_analysis.Gsea.1545201170987/HALLMARK_MYOGENESIS.html]

Details for gene set HALLMARK\_MYOGENESIS[GSEA]

|  || Dataset | expr.class.cls#HYDEGF\_versus\_CONTROL.class.cls#HYDEGF\_versus\_CONTROL\_repos |
| Phenotype | class.cls#HYDEGF\_versus\_CONTROL\_repos |
| Upregulated in class | HYDEGF |
| GeneSet | HALLMARK\_MYOGENESIS |
| Enrichment Score (ES) | 0.24991769 |
| Normalized Enrichment Score (NES) | 1.0923027 |
| Nominal p-value | 0.26666668 |
| FDR q-value | 0.41461062 |
| FWER p-Value | 1.0 |
Table: GSEA Results Summary

  

Fig 1: Enrichment plot: HALLMARK\_MYOGENESIS      
 Profile of the Running ES Score & Positions of GeneSet Members on the Rank Ordered List

  

| PROBE | DESCRIPTION (from dataset) | GENE SYMBOL | GENE\_TITLE | RANK IN GENE LIST | RANK METRIC SCORE | RUNNING ES | CORE ENRICHMENT || 1 | CKMT2 | na |  |  | 315 | 2.357 | 0.0042 | Yes |
| 2 | AEBP1 | na |  |  | 416 | 2.216 | 0.0184 | Yes |
| 3 | GADD45B | na |  |  | 524 | 2.096 | 0.0312 | Yes |
| 4 | FLII | na |  |  | 608 | 2.003 | 0.0445 | Yes |
| 5 | MYLK | na |  |  | 828 | 1.802 | 0.0488 | Yes |
| 6 | PYGM | na |  |  | 842 | 1.788 | 0.0639 | Yes |
| 7 | KIFC3 | na |  |  | 870 | 1.771 | 0.0780 | Yes |
| 8 | TEAD4 | na |  |  | 916 | 1.740 | 0.0909 | Yes |
| 9 | DTNA | na |  |  | 946 | 1.718 | 0.1045 | Yes |
| 10 | SPTAN1 | na |  |  | 1042 | 1.658 | 0.1141 | Yes |
| 11 | COL6A3 | na |  |  | 1059 | 1.646 | 0.1277 | Yes |
| 12 | MYO1C | na |  |  | 1516 | 1.425 | 0.1163 | Yes |
| 13 | MYH11 | na |  |  | 1734 | 1.347 | 0.1168 | Yes |
| 14 | PDE4DIP | na |  |  | 1763 | 1.340 | 0.1271 | Yes |
| 15 | COL1A1 | na |  |  | 1873 | 1.303 | 0.1328 | Yes |
| 16 | ENO3 | na |  |  | 1899 | 1.295 | 0.1429 | Yes |
| 17 | COL4A2 | na |  |  | 1989 | 1.269 | 0.1494 | Yes |
| 18 | ITGA7 | na |  |  | 2100 | 1.239 | 0.1545 | Yes |
| 19 | MAPK12 | na |  |  | 2157 | 1.224 | 0.1623 | Yes |
| 20 | COX7A1 | na |  |  | 2159 | 1.223 | 0.1730 | Yes |
| 21 | ATP2A1 | na |  |  | 2312 | 1.175 | 0.1754 | Yes |
| 22 | MYH3 | na |  |  | 2329 | 1.169 | 0.1848 | Yes |
| 23 | SMTN | na |  |  | 2459 | 1.139 | 0.1881 | Yes |
| 24 | KLF5 | na |  |  | 2481 | 1.132 | 0.1969 | Yes |
| 25 | AKT2 | na |  |  | 2780 | 1.056 | 0.1906 | Yes |
| 26 | TAGLN | na |  |  | 2945 | 1.017 | 0.1909 | Yes |
| 27 | DMPK | na |  |  | 2991 | 1.006 | 0.1974 | Yes |
| 28 | STC2 | na |  |  | 3009 | 1.002 | 0.2053 | Yes |
| 29 | PICK1 | na |  |  | 3206 | 0.957 | 0.2034 | Yes |
| 30 | ADAM12 | na |  |  | 3301 | 0.943 | 0.2068 | Yes |
| 31 | TPM2 | na |  |  | 3484 | 0.908 | 0.2052 | Yes |
| 32 | LAMA2 | na |  |  | 3609 | 0.883 | 0.2065 | Yes |
| 33 | SIRT2 | na |  |  | 3643 | 0.877 | 0.2124 | Yes |
| 34 | IFRD1 | na |  |  | 3686 | 0.868 | 0.2179 | Yes |
| 35 | NAV2 | na |  |  | 3798 | 0.848 | 0.2195 | Yes |
| 36 | TSC2 | na |  |  | 4032 | 0.807 | 0.2144 | Yes |
| 37 | BIN1 | na |  |  | 4034 | 0.806 | 0.2214 | Yes |
| 38 | CHRNG | na |  |  | 4052 | 0.804 | 0.2276 | Yes |
| 39 | VIPR1 | na |  |  | 4055 | 0.803 | 0.2345 | Yes |
| 40 | SPEG | na |  |  | 4116 | 0.794 | 0.2383 | Yes |
| 41 | LDB3 | na |  |  | 4242 | 0.776 | 0.2386 | Yes |
| 42 | NOTCH1 | na |  |  | 4284 | 0.769 | 0.2432 | Yes |
| 43 | FST | na |  |  | 4286 | 0.769 | 0.2499 | Yes |
| 44 | PSEN2 | na |  |  | 4592 | 0.722 | 0.2403 | No |
| 45 | PLXNB2 | na |  |  | 4893 | 0.669 | 0.2304 | No |
| 46 | ADCY9 | na |  |  | 4979 | 0.655 | 0.2317 | No |
| 47 | PDLIM7 | na |  |  | 5157 | 0.629 | 0.2279 | No |
| 48 | PFKM | na |  |  | 5189 | 0.622 | 0.2318 | No |
| 49 | SH2B1 | na |  |  | 5223 | 0.617 | 0.2355 | No |
| 50 | SCHIP1 | na |  |  | 5310 | 0.603 | 0.2363 | No |
| 51 | SORBS1 | na |  |  | 5377 | 0.593 | 0.2380 | No |
| 52 | LSP1 | na |  |  | 5501 | 0.576 | 0.2366 | No |
| 53 | CAV3 | na |  |  | 5542 | 0.571 | 0.2395 | No |
| 54 | SH3BGR | na |  |  | 5935 | 0.512 | 0.2235 | No |
| 55 | DMD | na |  |  | 5983 | 0.504 | 0.2255 | No |
| 56 | MRAS | na |  |  | 6023 | 0.499 | 0.2278 | No |
| 57 | SPHK1 | na |  |  | 6182 | 0.478 | 0.2237 | No |
| 58 | BAG1 | na |  |  | 6712 | 0.409 | 0.1996 | No |
| 59 | CACNA1H | na |  |  | 6843 | 0.391 | 0.1962 | No |
| 60 | NQO1 | na |  |  | 7079 | 0.362 | 0.1870 | No |
| 61 | EPHB3 | na |  |  | 7111 | 0.356 | 0.1885 | No |
| 62 | ABLIM1 | na |  |  | 7413 | 0.315 | 0.1755 | No |
| 63 | HDAC5 | na |  |  | 8276 | 0.212 | 0.1322 | No |
| 64 | TNNC1 | na |  |  | 8355 | 0.201 | 0.1298 | No |
| 65 | MAPRE3 | na |  |  | 8510 | 0.185 | 0.1234 | No |
| 66 | PPP1R3C | na |  |  | 8877 | 0.138 | 0.1054 | No |
| 67 | FHL1 | na |  |  | 8907 | 0.134 | 0.1051 | No |
| 68 | TCAP | na |  |  | 9067 | 0.119 | 0.0978 | No |
| 69 | CAMK2B | na |  |  | 9079 | 0.117 | 0.0982 | No |
| 70 | CHRNB1 | na |  |  | 9305 | 0.088 | 0.0872 | No |
| 71 | PPFIA4 | na |  |  | 9532 | 0.071 | 0.0760 | No |
| 72 | LPIN1 | na |  |  | 9566 | 0.067 | 0.0748 | No |
| 73 | COL15A1 | na |  |  | 9638 | 0.056 | 0.0716 | No |
| 74 | AK1 | na |  |  | 9668 | 0.052 | 0.0705 | No |
| 75 | MB | na |  |  | 10073 | 0.009 | 0.0494 | No |
| 76 | REEP1 | na |  |  | 10198 | -0.007 | 0.0430 | No |
| 77 | EIF4A2 | na |  |  | 10318 | -0.025 | 0.0370 | No |
| 78 | HBEGF | na |  |  | 10340 | -0.028 | 0.0361 | No |
| 79 | TPD52L1 | na |  |  | 10368 | -0.032 | 0.0350 | No |
| 80 | CRAT | na |  |  | 10668 | -0.062 | 0.0198 | No |
| 81 | ACSL1 | na |  |  | 11022 | -0.109 | 0.0023 | No |
| 82 | ITGB5 | na |  |  | 11075 | -0.118 | 0.0006 | No |
| 83 | ITGB4 | na |  |  | 11077 | -0.118 | 0.0016 | No |
| 84 | ST5 | na |  |  | 11232 | -0.128 | -0.0054 | No |
| 85 | SSPN | na |  |  | 12019 | -0.219 | -0.0447 | No |
| 86 | MYL6B | na |  |  | 12022 | -0.221 | -0.0429 | No |
| 87 | CKB | na |  |  | 12047 | -0.224 | -0.0422 | No |
| 88 | SVIL | na |  |  | 12087 | -0.231 | -0.0422 | No |
| 89 | ERBB3 | na |  |  | 12288 | -0.259 | -0.0504 | No |
| 90 | GNAO1 | na |  |  | 12648 | -0.305 | -0.0665 | No |
| 91 | SGCA | na |  |  | 13128 | -0.358 | -0.0885 | No |
| 92 | PC | na |  |  | 13181 | -0.366 | -0.0880 | No |
| 93 | SORBS3 | na |  |  | 13406 | -0.404 | -0.0962 | No |
| 94 | FDPS | na |  |  | 13646 | -0.433 | -0.1050 | No |
| 95 | TNNT1 | na |  |  | 13681 | -0.439 | -0.1029 | No |
| 96 | ACHE | na |  |  | 13812 | -0.461 | -0.1057 | No |
| 97 | MEF2D | na |  |  | 13861 | -0.468 | -0.1041 | No |
| 98 | SLC6A8 | na |  |  | 14163 | -0.502 | -0.1154 | No |
| 99 | TGFB1 | na |  |  | 14260 | -0.515 | -0.1160 | No |
| 100 | IGFBP7 | na |  |  | 14299 | -0.521 | -0.1134 | No |
| 101 | GAA | na |  |  | 14430 | -0.545 | -0.1154 | No |
| 102 | TNNC2 | na |  |  | 14460 | -0.549 | -0.1121 | No |
| 103 | COL6A2 | na |  |  | 14661 | -0.583 | -0.1175 | No |
| 104 | CDKN1A | na |  |  | 14882 | -0.616 | -0.1236 | No |
| 105 | RB1 | na |  |  | 15219 | -0.672 | -0.1353 | No |
| 106 | SYNGR2 | na |  |  | 15236 | -0.676 | -0.1302 | No |
| 107 | SOD3 | na |  |  | 15654 | -0.754 | -0.1454 | No |
| 108 | PTP4A3 | na |  |  | 15710 | -0.765 | -0.1416 | No |
| 109 | OCEL1 | na |  |  | 15738 | -0.772 | -0.1362 | No |
| 110 | CFD | na |  |  | 15870 | -0.795 | -0.1361 | No |
| 111 | HSPB8 | na |  |  | 16267 | -0.891 | -0.1491 | No |
| 112 | GSN | na |  |  | 16299 | -0.897 | -0.1428 | No |
| 113 | AGL | na |  |  | 16306 | -0.900 | -0.1352 | No |
| 114 | DAPK2 | na |  |  | 16407 | -0.925 | -0.1323 | No |
| 115 | PKIA | na |  |  | 16691 | -0.997 | -0.1384 | No |
| 116 | GPX3 | na |  |  | 16764 | -1.015 | -0.1333 | No |
| 117 | CNN3 | na |  |  | 16775 | -1.018 | -0.1249 | No |
| 118 | BHLHE40 | na |  |  | 16798 | -1.027 | -0.1170 | No |
| 119 | RIT1 | na |  |  | 16839 | -1.039 | -0.1099 | No |
| 120 | PRNP | na |  |  | 17039 | -1.101 | -0.1107 | No |
| 121 | SPDEF | na |  |  | 17163 | -1.131 | -0.1072 | No |
| 122 | ATP6AP1 | na |  |  | 17435 | -1.228 | -0.1106 | No |
| 123 | WWTR1 | na |  |  | 17497 | -1.258 | -0.1028 | No |
| 124 | TPM3 | na |  |  | 17535 | -1.272 | -0.0936 | No |
| 125 | MEF2C | na |  |  | 17565 | -1.283 | -0.0838 | No |
| 126 | FABP3 | na |  |  | 17863 | -1.422 | -0.0869 | No |
| 127 | MEF2A | na |  |  | 18278 | -1.660 | -0.0940 | No |
| 128 | CTF1 | na |  |  | 18460 | -1.806 | -0.0876 | No |
| 129 | SPARC | na |  |  | 18543 | -1.861 | -0.0756 | No |
| 130 | CLU | na |  |  | 18586 | -1.909 | -0.0610 | No |
| 131 | IGFBP3 | na |  |  | 18665 | -2.008 | -0.0474 | No |
| 132 | APP | na |  |  | 18704 | -2.052 | -0.0314 | No |
| 133 | FOXO4 | na |  |  | 18720 | -2.074 | -0.0140 | No |
| 134 | GABARAPL2 | na |  |  | 18751 | -2.113 | 0.0030 | No |
| 135 | SCD | na |  |  | 18875 | -2.317 | 0.0169 | No |
Table: GSEA details [plain text format]

  

Fig 2: HALLMARK\_MYOGENESIS      
 Blue-Pink O' Gram in the Space of the Analyzed GeneSet

  

Fig 3: HALLMARK\_MYOGENESIS: Random ES distribution      
 Gene set null distribution of ES for **HALLMARK\_MYOGENESIS**
